# Comparing antibiotic prescriptions in primary care between SARS-CoV-2 and influenza: a retrospective observational study

**DOI:** 10.3399/BJGPO.2022.0049

**Published:** 2022-11-16

**Authors:** Martijn Sijbom, Frederike L Büchner, Nicholas H Saadah, Mark GJ de Boer, Mattijs E Numans

**Affiliations:** 1 Department of Public Health and Primary Care, location Health Campus The Hague, Leiden University Medical Center, Leiden, The Netherlands; 2 Department of Infectious Diseases & Clinical Epidemiology, Leiden University Medical Center, Leiden, The Netherlands

**Keywords:** primary health care, SARS-CoV-2, antibiotics, influenza, human

## Abstract

**Background:**

Antibiotics are frequently prescribed during viral respiratory infection episodes in primary care. There is limited information about antibiotic prescription during the severe acute respiratory syndrome coronavirus 2 (SARS-CoV-2) pandemic in primary care and its association with risk factors for an adverse course.

**Aim:**

To compare the proportion of antibiotic prescriptions between patients with COVID-19 and influenza or influenza-like symptoms, and to assess the association between antibiotic prescriptions and risk factors for an adverse course of COVID-19.

**Design & setting:**

An observational cohort study using pseudonymised and coded routine healthcare data extracted from 85 primary care practices in the Netherlands.

**Method:**

Adult patients with influenza and influenza-like symptoms were included from the 2017 influenza season to the 2020 season. Adult patients with suspected or confirmed COVID-19 were included from the first (15 February 2020–1 August 2020) and second (1 August 2020–1 January 2021) SARS-CoV-2 waves. Proportions of antibiotic prescriptions were calculated for influenza and COVID-19 patients. Odds ratios (ORs) were used to compare the associations of antibiotic prescriptions in COVID-19 patients with risk factors, hospital admission, intensive care unit (ICU) admission, and mortality.

**Results:**

The proportion of antibiotic prescriptions during the first SARS-CoV-2 wave was lower than during the 2020 influenza season (9.6% versus 20.7%), difference 11.1% (95% confidence interval [CI] = 8.7 to 13.5). During the second SARS-CoV-2 wave, antibiotic prescriptions were associated with being aged ≥70 years (OR 2.05; 95% CI = 1.43 to 2.93), the number of comorbidities (OR 1.46; 95% CI = 1.18 to 1.82), and admission to hospital (OR 3.19; 95% CI = 2.02 to 5.03) or ICU (OR 4.64; 95% CI = 2.02 to 10.62).

**Conclusion:**

Antibiotic prescription was less common during the SARS-CoV-2 pandemic than during influenza seasons, and was associated with an adverse course and its risk factors. The findings suggest a relatively targeted prescription policy of antibiotics in primary care during COVID-19.

## How this fits in

Antibiotics are frequently prescribed during viral respiratory infection episodes in primary care to treat a presumed bacterial superinfection. This may also have occurred during the SAR-CoV-2 pandemic to treat COVID-19. To date, there is limited information on patterns of antibiotic prescription during the SARS-CoV-2 pandemic in primary care. This study shows antibiotics were less frequently prescribed in primary care during the SARS-CoV-2 pandemic compared with preceding influenza seasons. This likely points to more appropriate prescription of antibiotics when guided by dedicated diagnostic tests. Antibiotic prescription was associated with a more severe course of COVID-19, as would be expected. This suggests that GPs are able to estimate the risk of an adverse course.

## Introduction

The new SARS-CoV-2, like all viral respiratory tract infections (RTIs), carries a risk of bacterial superinfection.^1–3^ Antibiotics are often prescribed by GPs to reduce morbidity and mortality owing to these bacterial superinfections, particularly in the presence of certain risk factors.^1,4–7^ Influenza is a recognised major seasonal cause of viral RTIs and a trigger comparable with SARS-CoV-2 with regard to the risk of bacterial superinfections.^4^

There is limited information on the extent of antibiotic prescriptions in COVID-19 patients in primary care and the associations of these prescriptions with outcomes of interest. The main disadvantage of the use of antibiotics is the development of antimicrobial resistance (AMR).^8^ Another downside is the occurrence of potential side effects of antibiotics. Prudent antibiotic prescription is therefore still indicated and should be sustained in the current pandemic circumstances to reduce the risk of inappropriate antibiotic prescriptions to avoid unnecessary harm.

Antibiotic prescriptions were compared during recent influenza seasons with those of the first and second SARS-CoV-2 waves in The Netherlands. In addition, associations between antibiotic prescriptions and hospital admissions, ICU admissions, mortality, and various known risk factors were calculated.

## Method

### Study design and setting

#### Data collection

For this observational study, pseudonymised, coded routine healthcare data were used from patients enlisted between 2016 and 2020 with one of the 85 general practices participating in the Extramural LUMC Academic Network (ELAN) medical registry, operating out of the Leiden and The Hague area. GPs involved in this network provide complete and actively updated longitudinal data on their patients via their electronic medical records (EMRs). An informed opt-out procedure for the use of these pseudonymised data is in place.

### Inclusion

#### Influenza

Patients aged ≥18 years with influenza, upper RTIs, or flu-like symptoms were identified in the ELAN registry by searching the dossiers for the International Classification of Primary Care first edition (ICPC-1) codes (Table 1). Patients were included if they had any of these codes registered during influenza seasons 2017, 2018, 2019, or 2020 (Box 1).^9,10^

Box 1 **Definition and dates of influenza seasons studied^9,10^**

An influenza season is defined as more than 51 patients per 100 000 inhabitants with influenza-like illness or symptoms visiting their GP.

For season 2019–2020, the threshold was 58 patients per 100 000 inhabitants per week.

2017: 28 November 2016 up to including 6 March 2017.

2018: 11 December 2017 up to including 9 April 2018.

2019: 10 December 2018 up to including 11 March 2019.

2020: 27 January 2020 up to including 15 March 2020.

#### SARS-CoV-2

The following two definitions for diagnosis of a COVID-19 infection were accepted: (1) COVID-19 confirmed with a positive polymerase chain reaction (PCR) test and an appropriate ICPC code in the EMR (Table 1); and (2) COVID-19 highly suspected, based on symptoms (Box 2) and an appropriate ICPC code in the EMR (Table 1). The second definition was used owing to a lack of test capacity in The Netherlands from the start of the SARS-CoV-2 pandemic (February 2020) until 1 June 2020. Patients were included in the study if their PCR test or symptoms (Box 2) matched the definition of COVID-19, categorised as confirmed or suspected COVID-19, and divided in two groups according to their date of diagnosis.^11^ The first wave lasted from 15 February 2020–1 August 2020. The second wave lasted from 1 August 2020–1 January 2021. The SARS-CoV-2 index lineage was dominant in The Netherlands during both waves.^12^

**Table 1.** Overview of included ICPC-1 codes per group

ICPC-1 code	Influenza group	SARS-CoV-2 group
R74 Acute upper respiratory infection	Yes	Yes
R75 Acute/chronic sinusitis	Yes	Yes
R77 Acute laryngitis/tracheitis	Yes	Yes
R78 Acute bronchitis/bronchiolitis	Yes	Yes
R80 Influenza	Yes	Yes
R81 Pneumonia		Yes, excluding R81.01 Legionella pneumonia
R83 Other respiratory infection		Yes, excluding R83.01 Diphtheria and R83.02 Sarcoidosis

ICPC-1 = International Classification of Primary Care first edition. SARS-CoV-2 = severe acute respiratory syndrome coronavirus 2.

Box 2 **Symptoms of COVID-19^11^**

 Coughing

Cold

Sore throat

Shortness of breath while resting or during light exertion

Loss of taste or smell

Fever

Sudden fatigue

Diarrhoea

Headache

Conjunctivitis

Muscle and joint pains

**Table 2.** Definition of risk factors for adverse course of COVID-19

Risk factor	Definition and ICPC-1 codes where relevant
Age	Patients aged ≥70 years as of 1 January 2020
Sex	Male
Obesity	Body mass index >29 as of 1 January 2020
Smoking	Patents with an active or previous smoking status as of 1 January 2020
Heart disease^a^	K74 Angina pectoris, K75 and K76 Myocardial infarction, K77 Heart failure, K78 Atrial fibrillation
Diabetes mellitus^a^	T90 Diabetes mellitus
Severe chronic respiratory disease^a^	R91 Chronic bronchitis, R89 Congenital anomaly respiratory, R91 Bronchiectasis, R95 COPD
HIV infection^a^	B90 use of antiviral medication for HIV infection
Severe renal disease^a^	U99 (.01) Renal impairment and eGFR <25 ml/min/1.73 m^2^
Severe liver disease^a^	D97 Cirrhosis, Liver failure of liver decompensation, Contraindication label liver impairment
Down's syndrome^a^	A90 (.01) Down's syndrome

^a^These risk factors were merged into one comorbidity variable. The presence of each single risk factor or disease was counted as one and added together as count variable. COPD = chronic obstructive pulmonary disease. eGFR = estimated glomerular filtration rate. ICPC-1 = International Classification of Primary Care codes first edition.

#### Antibiotic prescriptions

The Anatomical Therapeutic Chemical Classification System code J01 was used to identify and extract data on oral antibiotic prescriptions from the ELAN registry. Prescriptions were linked with patients with influenza and patients with COVID-19 through the pseudonymised patient numbers following a check that the date of the antibiotic prescription corresponded with the registration date of the ICPC-1 code. If the date of the antibiotic prescription and the registration date did not correspond, the antibiotic prescription was not included.

#### Hospital and intensive care admissions and mortality

An adverse course of COVID-19 was defined in the study as a hospital admission, ICU admission, or mortality. Data on this adverse course were extracted from the EMR in the ELAN registry through examination of the free text in the EMR of each patient with COVID-19.

#### Risk factors for an adverse course of COVID-19

Risk factors tested for association with a severe course of COVID-19 were based on the definition by the Dutch National Institute for Public Health and the Environment (Rijksinstituut voor Volksgezondheid en Milieu; RIVM) and outcomes of recent literature reviews on risk factors for an adverse course of COVID-19.^13–15^ Included risk factors were as follows: age, sex, obesity, smoking, heart disease, diabetes mellitus, severe chronic respiratory disease, HIV infection, severe renal disease, severe liver disease, and Down's syndrome. The definitions are listed in Table 2.

### Outcome

The outcome measures were as follows: (a) number of antibiotic prescriptions and (b) proportion of patient contacts resulting in antibiotic prescriptions during influenza seasons 2017–2020 and during the two waves of the SARS-CoV-2 pandemic (2020); (c) the number of hospital admissions; (d) ICU admissions; and (e) deaths among patients with COVID-19.

### Statistical analysis

For comparison of extent of antibiotic prescription between SARS-CoV-2 waves and influenza seasons, the number of antibiotic prescriptions and proportion of patient contacts resulting in antibiotic prescriptions were compared via unpaired *t*-tests. Association testing between risk factors and outcome measures was performed using multivariate logistic regression with age, sex, obesity, and smoking added to the model as covariates with the additional risk factors, heart disease, diabetes mellitus, severe chronic respiratory disease, HIV infection, severe renal disease, severe liver disease, and Down's syndrome, merged into a composite comorbidity variable. For calculation of this composite variable, the presence of each risk factor or disease was counted as one and added together as a count variable. The multivariate logistic regression model tested the associations between these risk factors and outcome measures (a and b) antibiotic prescriptions, (c) hospital admissions, (d) ICU admissions, and (e) mortality.

Multiple imputation was used to address missing data for risk factors smoking and obesity. The imputation model included all covariates and outcomes (details of multiple imputation model in supplement 1). SPSS statistics (version 25) was used for statistical analysis.

## Results

In total, 1702 patients were diagnosed by their GP with suspected or confirmed COVID-19 in the first wave of 2020 with 6904 patients diagnosed in the second wave (Table 3). The total number of antibiotic prescriptions was similar during the first wave compared with the second wave (209 versus 238 prescriptions, respectively). The proportion of antibiotic prescriptions per patient contact was higher during the first wave, 9.6% (95% CI = 7.9 to 11.4), than during the second wave 2.7% (95% CI = 1.4 to 4.0). Influenza season 2020 had the lowest number of antibiotic prescriptions per contact (20.7%) of any influenza season analysed in the study. This was higher than during the first and second SARS-CoV-2 waves 9.6% (95% CI = 7.9 to 11.4) and 2.7% (95% CI = 1.4 to 4.0), respectively (Table 4). All influenza seasons had a higher proportion of antibiotic prescriptions per patient contact compared with both SARS-CoV-2 waves (Table 4). During the second wave, a higher proportion of the patients with suspected COVID-19 were prescribed antibiotics, 5.0% (95% CI = 3.8 to 6.2), compared with patients with confirmed COVID-19, 2.5% (95% CI = 1.3 to 3.7). During, the first wave, the proportion of prescribed antibiotics per contact was for patients with suspected, or confirmed COVID-19, 10.7% (95% CI = 7.8 to 13.6) and 6.1% (95% CI = 3.9 to 9.0), respectively.

**Table 3.** Patient characteristics

Diagnosis		Influenza				SARS-CoV-2	
Year or season		2017	2018	2019	2020	First wave	Second wave
Population size^a^		254 586	276 275	288 703	288 305	288 305	288 305
Number of patients		4579	8016	4354	1422	1702	6904
Age range in years (mean)		18–100 (51)	18–102 (51)	18–101 (51)	18–99 (48)	18–100 (50)	18–100 (48)
Confirmed COVID-19 (*n*)		–	–	–	–	247	5682
Suspected COVID-19 (*n*)		–	–	–	–	1455	1222
Number of contacts with GP practices		4858	9298	4922	1542	2165	8867
Risk factors for adverse course of COVID-1							
	Age ≥70 year % (*n*)	18.8 (860)	18.2 (1457)	18.5 (804)	13.3 (189)	14.9 (253)	11.7 (806)
	Male % (*n*)	35.4 (1622)	36.5 (2929)	34.6 (1507)	37.7 (536)	38.4 (653)	42.3 (2923)
	Obesity, BMI >29% (*n*)^b^	17.6 (807)	18.2 (1456)	18.9 (823)	17.2 (245)	6.6 (113)	16.6 (1147)
	Smoking: current and previous % (*n*)^c^	25.9 (1185)	25.9 (2077)	25.2 (1099)	23.1 (329)	9.8 (166)	19.3 (1330)
	Heart disease % (*n*)^d^	12.3 (565)	10.5 (844)	10.4 (452)	7.2 (102)	3.5 (59)	8.0 (550)
	Diabetes mellitus % (*n*)^e^	10.4 (477)	10.5 (839)	9.8 (427)	8.2 (116)	10.6 (181)	9.9 (682)
	Severe chronic respiratory disease (*n*)^f^	3.4 (154)	3.5 (277)	3.4 (150)	2.8 (40)	6.2 (105)	2.9 (198)
	HIV infection % (*n*)^g^	0.3 (13)	0.3 (21)	0.3 (15)	0.1 (1)	0.4 (6)	0.3 (20)
	Severe kidney disease (eGFR <26) % (*n*)^h^	0.4 (19)	0.4 (35)	0.2 (9)	0.2 (3)	0.6 (11)	0.3 (21)
	Liver failure % (*n*)^i^	0.1 (1)	0	0.1 (1)	0	0	0
	Down's syndrome % (*n*)	0.1 (1)	0.1 (1)	0.1 (3)	0	0	0.1 (1)

^a^ In total, 348 553 individual patients were registered during the study period 2016–2020 in the ELAN Datawarehouse. The population size per year is the number of patients registered during that study year. ^b^Missing BMI (year or season, *n*): 2017, 2507; 2018, 4338; 2019, 2378; 2020, 847; first wave, 1434; second wave, 4274. ^c^Missing smoke status (year or season, *n*): 2017, 2403; 2018, 4201; 2019, 2312; 2020, 805; first wave, 1404; second wave, 4182. ^d^Heart disease: ICPC K74 Angina pectoris, ICPC K75 and K76 Myocardial infarction, ICPC K77 Heart failure, ICPC K78 Atrial fibrillation.^e^Diabetes mellitus: ICPC T90 Diabetes mellitus. ^f^Severe chronic respiratory disease: ICPC R91 Chronic bronchitis, ICPC R89 Congenital anomaly respirator, ICPC R91 Bronchiectasis, ICPC R95 COPD.^g^HIV infection: ICPC B90 Use of antiviral medication for an HIV infection.^h^Severe renal disease: ICPC U99 (.01) Renal impairment and eGFR <25 ml/min/1.73 m^2^. ^I^Liver failure: ICPC D97 Cirrhosis, Liver failure of liver decompensation, Contraindication label liver impairment.

BMI = body mass index. ICPC = International Classification of Primary Care codes first edition. SARS-CoV-2 = severe acute respiratory syndrome coronavirus 2.

**Table 4.** Number of antibiotic prescriptions per season per group and observed outcome

Diagnosis		Influenza				SARS-CoV-2	
Year or season		2017	2018	2019	2020	First wave	Second wave
Number of patients		4579	8016	4354	1422	1702	6904
Number of contacts with GP practices		4858	9298	4922	1542	2165	8867
Antibiotic prescriptions per total contacts % (*n*)		25.1 (1221)	27.9 (2595)	29.6 (1458)	20.7 (319)	9.6 (209)	2.7 (238)
	Penicillins % (*n*)	13.9 (676)	15.7 (1458)	17.7 (869)	12.6 (194)	6.7 (145)	2.0 (177)
	Macrolides % (*n*)	3.0 (147)	3.9 (364)	3.7 (184)	2.5 (38)	1.0 (21)	0.3 (27)
	Tetracyclines % (*n*)	8.1 (393)	8.1 (755)	8.1 (397)	5.5 (85)	1.7 (37)	0.3 (30)
	Other % (*n*)	0.1 (5)	0.2 (18)	0.1 (8)	0.1 (2)	0.3 (6)	0.1 (4)
Observed outcome							
	Hospital admissions % (*n*)	–	–	–	–	7.5 (128)	3.3 (227)
	Intensive care admissions % (*n*)	–	–	–	–	1.5 (25)	0.6 (41)
	Mortality % (*n*)	–	–	–	–	2.1 (36)	1.0 (71)
Difference in proportion of antibiotic prescriptions between influenza seasons and SARS-CoV-2 waves							
	First wave % (95% CI)	15.5 (13.8 to 17.2)	18.3 (16.8 to 19.8)	20.0 (18.2 to 21.8)	11.1 (8.7 to 13.5)	–	–
	Second wave % (95% CI)	22.4 (21.1 to 23.7)	25.2 (24.2 to 26.2)	26.9 (25.6 to 28.2)	18.0 (15.9 to 20.1)	–	–

SARS-CoV-2 = severe acute respiratory syndrome coronavirus 2.

**Figure 1.**
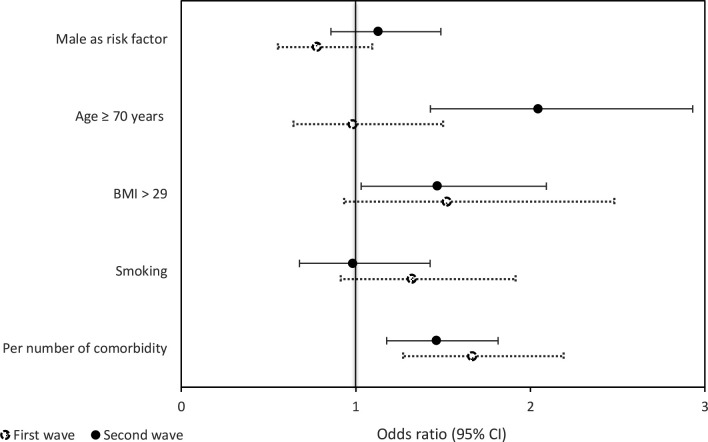
Risk factors associated with receiving an antibiotic prescription. BMI = body mass index. CI = confidence interval. Multivariate logistic regression was performed with pooled imputed data and outcomes were adjusted for all risk factors.

**Figure 2.**
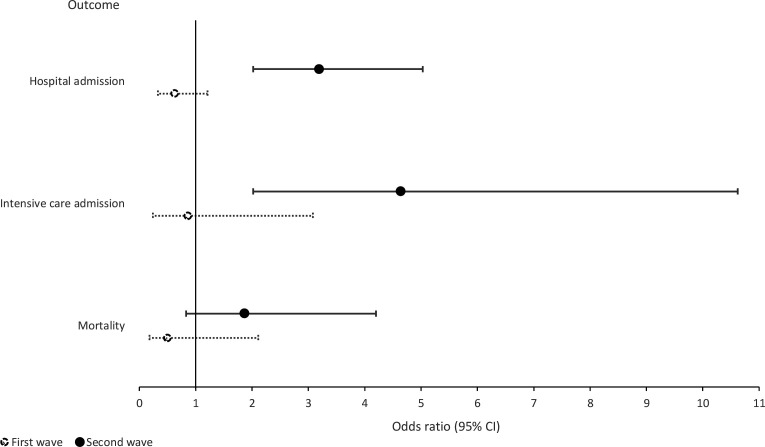
Observed outcome after antibiotic prescription for SARS-CoV-2. BMI = body mass index. CI = confidence interval. Multivariate logistic regression was performed with pooled imputed data and outcomes were adjusted for all risk factors.

Similar effect estimates were found with multivariate logistic regression using original or pooled imputed data. Therefore, results from multivariate logistic regression with pooled imputed data are presented. During the second wave, an antibiotic prescription was positively associated with an age of ≥70 years (OR 2.05; 95% CI = 1.43 to 2.93), the number of comorbidities (OR 1.46; 95% CI = 1.18 to 1.82) (Figure 1), a hospital admission (OR 3.19; 95% CI = 2.02 to 5.03) or ICU admission (OR; 4.64 95% CI = 2.02 to 10.62) (Figure 2).

## Discussion

### Summary

In this study, the frequencies of antibiotic prescription during SARS-CoV-2 episodes were compared with those of preceding influenza episodes. Antibiotic prescriptions were found to be less frequently used in primary care during SARS-CoV-2 waves than during influenza seasons 2017 up to and including 2020. Antibiotic prescriptions during the second SARS-CoV-2 wave were associated with older age, the number of comorbidities, and also with hospital or ICU admission later. This association was not observed during the first wave.

### Comparison with existing literature

In the study population, antibiotics were prescribed for 20–30% of patients with influenza-like illness or influenza. This may, according to the guidelines, be interpreted as inappropriate prescription. Other Dutch studies likewise show excessive antibiotic prescription during viral RTI episodes by GPs.^6,16,17^ However, these studies include different symptoms and diseases, which makes them difficult to compare directly. The prescription of antibiotics was less common during the SARS-CoV-2 pandemic in The Netherlands compared with the rates recorded for RTIs pre-SARS-CoV-2.

The proportion of antibiotic prescriptions per contact for COVID-19 during the first wave (9.6%) was comparable with antibiotic prescribing in the management of RTI symptoms in Dutch primary care reported in a study of van der Velden *et al* during the SARS-CoV-2 pandemic (7.1%).^18^

In the present study, the total sum of antibiotic prescriptions during SARS-CoV-2 did not differ much between the first and second waves. This, in spite of the burden of the SARS-CoV-2 pandemic being higher during the second compared with the first wave, reflected by the higher number of hospital admissions for COVID-19 patients in The Netherlands.^19^ The relatively higher frequency of antibiotic prescriptions during the first wave may partly be owing to registration bias, as not all COVID-19 patients during the first wave were registered. Another reason for the less frequent prescription of antibiotics during the second wave may be the increasing knowledge on disease course and risk factors for severe deterioration of COVID-19. Further, there were fewer non-COVID RTIs during the SARS-CoV-2 pandemic.^20^ The high probability of a SARS-CoV-2 infection combined with accessible PCR testing aids the GP with diagnostic accuracy and likely decreases antibiotic prescription.

### Strengths and limitations

A strength of the study is the comparison of antibiotic prescriptions during influenza seasons with those during the SARS-Cov-2 pandemic. Influenza was already a major seasonal cause of viral RTIs and antibiotic prescriptions, and now SARS-CoV-2, at least initially, may have the same effect on GPs' prescribing behaviour in primary health care. Influenza patients and patients with COVID-19 present with similar symptoms. Therefore, the initial assessment does not differ between the two diseases. However, the study revealed increasing differences in antibiotic prescriptions, which may reflect increasing experience among physicians in judging disease severity, or better estimates of potential adverse disease course development.

The results of the study may be hindered by registration bias as not all COVID-19 patients were registered (correctly) before 1 June 2020. The gold standard for diagnosing COVID-19 patients is a positive PCR test from a nasal and throat swab.^10^ Until 1 June 2020, there was a lack of PCR-testing capacity in The Netherlands. As a consequence, only patients with COVID-19 symptoms assessed at an emergency department were tested. Until June 1 2020, GPs mainly based a COVID-19 diagnosis on the medical history, patient characteristic, and reported and observed symptoms. Patients were advised to contact their GP if they experienced severe symptoms. This led to under-registration of COVID-19 patients in the first wave, leading to a higher proportion of patients with a severe course of COVID-19 being registered. From June 2020 onwards, all patients with symptoms could be tested for SARS-CoV-2 by the municipal health services and test results were quickly passed on to GPs. But patients could have to wait up to 3 days before a PCR test was performed and the results were passed on. Meanwhile, they may have contacted their GPs, leading to a registration of suspected COVID-19.

At the start of the SARS-CoV-2 pandemic in The Netherlands, patients with (suspicion of) COVID-19 were not uniformly registered in the EMR with the same ICPC code. A separate ICPC code, R83.03 SARS-CoV-2, was introduced in November 2020, and slowly implemented. Most patients were registered according to their 'influenza-like' symptoms. For this reason, patients aged ≥18 years with the ICPC codes listed in Table 1 were selected broadly from the study population. As only respiratory ICPC codes were selected, asymptomatic patients with COVID-19 or patients with only non-respiratory symptoms associated with SARS-CoV-2 were potentially missed. Use of routinely collected healthcare data always carries a risk of missing data, as was the case in the present study. The authors feel confident missing data in the study is missing at random. The percentage of hospital admissions and mortality during the second wave were comparable with national percentages, suggesting any selection and registration bias in the second wave was low.^12,21^ As such, the analysis of the second wave was addressed in the primary discussion.

### Implications for research and practice

It was found antibiotic prescriptions were given less often during SARS-CoV-2 waves compared with influenza seasons. This may be owing to proper testing of patients for COVID-19, along with a coinciding lower prevalence of influenza and other respiratory viruses, leading to less diagnostic uncertainty about potentially missing a bacterial infection. This may have led to more confidence in the diagnostic accuracy among physicians and hence to communicating a diagnosis to a patient with more certainty.

As a result, antibiotics to prevent or treat a possible bacterial superinfection were largely restricted to those assessed to be at risk of developing or having a more adverse course of COVID-19. Since COVID-19 testing might be the most probable explanation of increased appropriateness in antibiotic prescriptions over time, rapid point-of-care tests for influenza and other viral RTIs may further reduce diagnostic uncertainty and result in fewer antibiotic prescriptions during viral RTI episodes. A Dutch study in primary care has already suggested that point-of-care testing for patients with RTIs may decrease antibiotic prescriptions.^22^

In conclusion, this study confirmed that a high proportion of patients with influenza in the past four seasons were treated with antibiotics by their GP. In contrast, the rate of antibiotic prescription in primary care during the first two waves of the SARS-CoV-2 pandemic in The Netherlands was lower than the influenza seasons studied. Patients with COVID-19 who were prescribed an antibiotic were more likely to have risk factors and more often experienced an adverse course of COVID-19, as is shown by an increased number of hospital or ICU admissions among those prescribed antibiotics. These observations suggest a relatively targeted antibiotic prescription policy during COVID-19, but also clearly suggest that inappropriate antibiotic prescription would potentially decrease further with diagnostic testing for other specific viral infections.

## Data

A set with coded routine Medical Record data, pseudonymized and extracted from primary care practices towards the ELAN datawarehouse was used for the analysis of antibiotic prescriptions during influenza and SARS-CoV-2 seasons. This dataset cannot be shared in an open public repository. Medical data in the ELAN datawarehouse are pseudonymized, so theoretically patients still can be identified and confidentiality could be violated. Patients did consent to reuse their medical data for the purpose of dedicated and contextually restricted research and quality management, but not in an open and publicly available domain. So, data are available upon reasonable request at the ELAN datawarehouse through elanresearch.nl.
